# Measurement variability of blood–brain barrier permeability using dynamic contrast-enhanced magnetic resonance imaging

**DOI:** 10.1162/imag_a_00324

**Published:** 2024-10-22

**Authors:** Aravinthan Varatharaj, Carmen Jacob, Angela Darekar, Brian Yuen, Stig Cramer, Henrik Larsson, Ian Galea

**Affiliations:** Clinical Neurosciences, Clinical and Experimental Sciences, Faculty of Medicine, University of Southampton, Southampton, United Kingdom; Wessex Neurological Centre, University Hospital Southampton NHS Foundation Trust, Southampton, United Kingdom; Medical Physics, University Hospital Southampton NHS Foundation Trust, Southampton, United Kingdom; Medical Statistics, Primary Care, Population Sciences and Medical Education, Faculty of Medicine, University of Southampton, Southampton, United Kingdom; Functional Imaging Unit, Department of Clinical Physiology and Nuclear Medicine, Rigshospitalet, Glostrup, Denmark

**Keywords:** dynamic contrast-enhanced magnetic resonance imaging, blood–brain barrier, measurement variability, scan–rescan

## Abstract

Dynamic contrast-enhanced magnetic resonance imaging (DCE-MRI) is used to quantify the blood–brain barrier (BBB) permeability–surface area product. Serial measurements can indicate changes in BBB health, of interest to the study of normal physiology, neurological disease, and the effect of therapeutics. We performed a scan–rescan study to inform both sample size calculation for future studies and an appropriate reference change value for patient care. The final dataset included 28 healthy individuals (mean age 53.0 years, 82% female) scanned twice with mean interval 9.9 weeks. DCE-MRI was performed at 3T using a 3D gradient echo sequence with whole brain coverage, T1 mapping using variable flip angles, and a 16-min dynamic sequence with a 3.2-s time resolution. Segmentation of white and grey matter (WM/GM) was performed using a 3D magnetization-prepared gradient echo image. The influx constant K_i_ was calculated using the Patlak method. The primary outcome was the within-subject coefficient of variation (CV) of K_i_ in both WM and GM. K_i_ values followed biological expectations in relation to known GM/WM differences in cerebral blood volume (CBV) and consequently vascular surface area.

Subject-derived arterial input functions showed marked within-subject variability which were significantly reduced by using a venous input function (CV of area under the curve 46 vs. 12%, p < 0.001). Use of the venous input function significantly improved the CV of K_i_ in both WM (30 vs. 59%, p < 0.001) and GM (21 vs. 53%, p < 0.001). Further improvement was obtained using motion correction, scaling the venous input function by the artery, and using the median rather than the mean of individual voxel data. The final method gave CV of 27% and 17% in WM and GM, respectively. No further improvement was obtained by replacing the subject-derived input function by one standard population input function. CV of K_i_ was shown to be highly sensitive to dynamic sequence duration, with shorter measurement periods giving marked deterioration especially in WM. In conclusion, measurement variability of 3D brain DCE-MRI is sensitive to analysis method and a large precision improvement is obtained using a venous input function.

## 1 Introduction

Dynamic contrast-enhanced MRI (DCE-MRI) quantifies blood–brain barrier (BBB) permeability to an intravenous bolus of gadolinium-based contrast agent. Changes in T1 relaxation time are related to contrast concentration measured in a tissue of interest and in a feeding vessel (input function). Tracer kinetic analysis of concentration–time curves derives the influx constant K_i_, an index of blood-to-brain transfer rate. According to the Crone–Renkin equation, K_i_ approximates the permeability–surface area product when the extraction fraction is low, as is the case for gadolinium contrast in the healthy brain or in neurological disease featuring subtle BBB disruption. We have previously validated our technique as fulfilling the expectations of K_i_ in relation to cerebral blood volume (CBV), cerebral blood flow (CBF), and tissue type (grey/white matter) (Varatharaj et al., 2019). Our group has employed DCE-MRI for predicting multiple sclerosis (MS) risk (Cramer et al., 2015) and treatment response (Cramer et al., 2018; Knudsen et al., 2022), and many other groups have shown uses in dementia and neurological conditions (Montagne et al., 2022; Voorter et al., 2024).

Despite this, DCE-MRI has barriers to adoption in clinical practice or as an outcome in clinical trials. One barrier is the relative lack of data on measurement variability (Thrippleton, 2019), which poses challenges both for sample size calculation in trials and for interpretation of serial measurements in patient care. Variability is inherent in any measurement. Serial values of any biomarker within a subject will not be identical due to (1) *analytical imprecision*: variability in the method itself and (2) *biological variation*: changes in the ground truth value occurring in the absence of any change in health or disease state.

One approach is to study analytical imprecision using a phantom, in which the effects of biological variation are removed (Freed et al., 2011). However, not all sources of imprecision operate within a phantom, and, therefore, it is difficult to apply the findings to *in vivo* measurements.

A scan–rescan study can be used to calculate measurement variability, and when the interval between scans is very short (i.e., in the same session), it is assumed that the effect of biological variability is minimised, and analytical imprecision dominates. However, this value alone would be of limited use for clinical trials or patient care, where the inter-scan interval is likely to be much longer, with a greater component of biological variation; we do not have good data on biological variation in BBB permeability (by DCE-MRI on any other method). Our approach is pragmatic; whilst it would be ideal to quantify the relative contribution of analytical and biological components to total variability, instead we study total within-subject scan–rescan variation in a population and time frame relevant to a typical study using BBB permeability change as the primary outcome.

The aims of this study are, therefore, to (1) measure within-subject variability (reflecting both analytical and biological components) over an 8-week interval and (2) test which acquisition and post-processing parameters minimise this variability whilst fulfilling expectations of K_i_ in relation to CBV, CBF, and tissue type.

## 2 Methods

### 2.1 Participants

Thirty healthy adult individuals were recruited by advertisement. Inclusion criteria were as follows: age 40–80 years, no systemic or neurological disease (including active migraine), no regular medication use, and no family history of MS. All subjects were examined by a neurologist prior to inclusion. Baseline demographic and clinical data were collected at inclusion. The age range was chosen to reflect a population relevant to our clinical practice and trials; younger individuals might have different biological variation in BBB permeability.

Participants were scanned in two separate sessions as close as possible to 8 weeks apart. The time frame was selected as one over which, in a clinical setting, one might anticipate a treatment- or disease-related effect. First and second scans were performed in an interleaved fashion to prevent the possibility of systematic bias due to longitudinal scanner signal drift. In the event of an intercurrent infection, illness, or vaccination following the first scan, the second scan was delayed by at least 6 weeks post-event or recovery to prevent confounding by a possible biological effect.

The study was approved by the National Research Ethics Service Committee London Surrey (reference 18/LO/2015) and the institutional review board (ERGO 46018). Experiments were conducted in accordance with the Declaration of Helsinki and all subjects gave informed written consent.

### 2.2 MR acquisition protocol

Imaging was performed on a 3 T MR unit (Skyra, Siemens, Erlangen, Germany) using a 20‐element phased‐array head coil. The dynamic sequence comprised 3D gradient echo (TR 2.48 ms, TE 0.99 ms, flip angle 15°, linear phase ordering, GRAPPA with parallel imaging factor 2, acquired matrix 192 × 144 × 30, field-of-view 250 × 188 × 150 mm^3^, voxel size 1.3 × 1.3 × 5.0 mm^3^, reconstructed into 30 slices of thickness 5.0 mm, 300 frames, time resolution 3.2 s, total scan duration 16 min). We found no significant evidence of scanner drift over this measurement period in both phantom and human experiments. Contrast agent was Gadovist (Bayer, Newbury, UK) given as a split bolus of 0.05 mmol kg^−1^ at 10th and 45th timepoints with automated intravenous injector (Medrad, Newbury, UK) at a rate of 3 ml s^-1^ followed by 30 ml saline flush. The rationale for a double bolus was to prevent signal truncation due to possible peak-dose T2* effects, as well as reduce the effect of input function temporal mis-sampling (Roberts et al., 2006). Baseline T1 mapping used the same sequence as above with TR = 10 ms, number of excitations averaged = 4, and variable flip angles (5, 15°) chosen to optimise accuracy and precision over the anticipated T1 range (Cheng & Wright, 2006) (in preliminary work we found no benefit with a third intermediate flip angle). A B1 map was acquired with the slice-selective preparation pulse method (Chung et al., 2010), with the same coverage as the T1 mapping sequence but acquired matrix 64 x 64 x 15. Prior to the dynamic acquisition, we also performed a 3D magnetisation-prepared gradient echo (MP-RAGE) sequence (TR = 2,200 ms, TE = 2.45 ms, TI = 900 ms, flip angle = 8°, field-of-view 263 × 350 × 350 mm^3^, voxel size 1.0 × 1.0 × 1.0 mm^3^).

### 2.3 DCE analysis protocol

Tracer kinetic analysis with the Patlak model (Patlak et al., 1983) was performed using custom code in MATLAB (Mathworks, Natick, USA) as previously described (Varatharaj et al., 2019), to derive estimates of K_i_ and CBV (as the slope and intercept of the Patlak plot, respectively). CBF estimation was done using model-free deconvolution of the tissue concentration with the arterial input function (Larsson et al., 2008).

K_i_ measured in whole blood can be converted to plasma K_trans_, preferred by recent guidelines (Dickie et al., 2024), by correction using the haematocrit. However, measurement of the haematocrit adds an additional source of variability, and, when sampled from a large vein, is not representative of microvessel haematocrit (Calamante et al., 2016). Alternatively, if a fixed value for the haematocrit is used, as in many studies, then K_i_ and K_trans_ simply scale. Therefore, in this study K_i_ was used.

At each timepoint, WM/GM probability maps were generated from the MP-RAGE using FSL-FAST (Zhang et al., 2001), transformed to DCE-MRI space, and a threshold applied such that only voxels with at least 95% probability of classification to the selected tissue were included in the resulting mask. Voxels with more than 0% probability of classification as cerebrospinal fluid (CSF) were excluded. Manual quality control showed that this procedure operated rigorously throughout.

Several additional analysis factors were tested:

- *Motion correction* (default = off): Two-step rigid registration of all dynamic frames to first T1 mapping image, via high-resolution MP-RAGE, using linear registration (FSL-FLIRT, FMRIB) (Jenkinson et al., 2002).
- *B1 mapping* (default = off): The acquired B1 map was interpolated by cubic spline to match T1 mapping geometry and filtered with a 3D Gaussian kernel (σ = 4.2, size = 10 mm) within a skull-stripped brain mask. Voxel-wise B1 values were used to correct flip angle values both during T1 mapping and in the conversion of dynamic signal to concentration.
- *Correction for incomplete spoiling* (default = off): The efficiency of spoiling in a gradient echo sequence becomes compromised when using a very short TR (Preibisch & Deichmann, 2009), as is commonly the case in DCE-MRI. A published correction method was used to compensate, both in T1 mapping and in conversion of dynamic signal to concentration (Baudrexel et al., 2018).
- *Input function location* (default = artery): The arterial input function was derived from the supraclinoid segment of the internal carotid artery (ICA), whilst the venous input function was derived from the posterior segment of the superior sagittal sinus (SSS) near the torcula. Use of the vein is recommended by consensus guidelines due to reduction of partial volume, inflow, and motion artefacts (Thrippleton et al., 2019).
- *Input function method* (default = manual): For the manual method, the input function region of interest (ROI) was created by a single operator (A.V.). Examples are shown in Supplementary Figure 1. For the automated method, we first generated standard binary masks of the supraclinoid ICA and posterior SSS using high-resolution atlases of brain arterial (Forkert et al., 2013) and venous (Huck et al., 2019) anatomy, respectively. For each scan, the automated pipeline then used a two-step process to transform each mask from standard space to subject MP-RAGE space using FSL’s non-linear registration tool FNIRT (Andersson, Jenkinson, & Smith), and then to dynamic space using FLIRT (Jenkinson et al., 2002). The signal–time curves of all voxels within either artery or vein mask were then evaluated according to published criteria of area under the curve and roughness (Mouridsen et al., 2006) to delineate an input function ROI.
- *Input function scaling* (default = off): Assuming negligible uptake, arterial signals were scaled by venous signal to account for inflow artefact, according to a published method (Hansen et al., 2009). Venous signals were time shifted to match the arterial bolus peak according to the same method.
- *Averaging level* (default = signal-wise): For the signal-wise method, the average signal for all voxels in the tissue mask was converted to concentration and passed to model fitting, whereas for the parameter-wise method, concentration–time curves and tracer kinetic models were fitted for each individual voxel in the mask, and the resulting parameters averaged. Representative voxel-wise parameter maps are shown in Supplementary Figure 2.
- *Averaging method* (default = mean): Either mean or median.

Since the effect of factors may interact, a factorial analysis was conducted. With all possible factor combinations, 2^8^ = 256 unique analysis variants were tested for each scan. The “default” analysis (as outlined above) was used as a comparator, and is the same as we used in a previous study (Varatharaj et al., 2019).

An *a priori* decision was made regarding negative K_i_ values, which sometimes arise in Patlak fitting. Random noise can generate a positive as well as a negative K_i_. If the true value of BBB permeability is close to zero (as is likely in the healthy brain), then random noise could give rise to negative K_i_ values. Negative values can also arise if contrast effluxes back into the intravascular space (violating the Patlak assumption of irreversible trapping), as this would bias K_i_ towards lower values and, therefore, increase the likelihood of negative values due to superimposed noise. In other studies, negative values have been removed by a variety of methods (Huisa et al., 2015; Wong et al., 2017). Since the object of this study was to account for analytical imprecision including random noise components, no positivity constraint was applied to the data (the proportion of voxels with negative K_i_ values is shown in Supplementary Table 1).

To separate the effect of input function variability, the highest ranked analysis was re-run but for each individual subject replacing the subject-derived input function with one standard input function derived from an external population (Parker et al., 2006), which was used for all subjects at both timepoints. Whether this population-derived input function is more representative of the true input function for each individual is debatable, but by definition, this removes any within-subject variability in the measurement of a subject-derived input function.

**Fig. 1.**
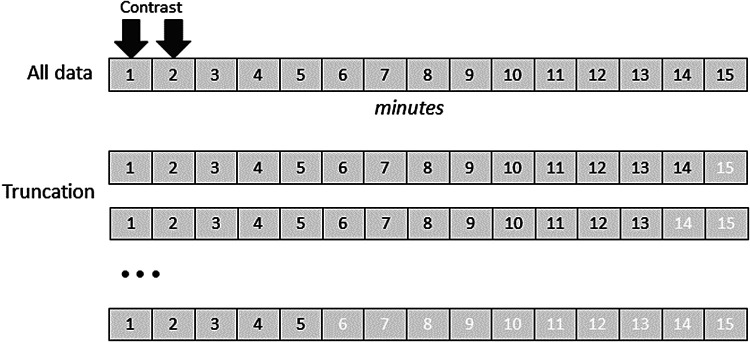
Illustration of how data were truncated to test the effect of dynamic sequence duration.

To simulate the effect of DCE protocols with shorter post-contrast acquisition times, the highest ranked analysis was re-run but truncating the data to 1-min increments between 5 and 16 min (see Fig. 1) and reporting the effect on K_i_ and its coefficient of variation (CV).

### 2.4 Statistics

All analyses were reported for white matter (WM) and grey matter (GM) separately.

Absolute K_i_ values were reported by taking the mean of both timepoints as the per-subject value. Results from each analysis were tested against the criteria previously described for the biological expectations of K_i_ (Varatharaj et al., 2019), namely:

1. Higher K_i_ in GM versus WM, tested in a paired *t*-test and examining the within-subject GM/WM ratio.
2. Dependence of K_i_ on CBV, tested in a step-wise multiple regression of K_i_ against CBV, CBF, and tissue type (for each continuous variable, taking the mean of both timepoints as the per-subject value).
3. No relationship between K_i_ and CBF, tested in the above regression (if CBF explained no variance in addition to CBV, it was not included in the model).

The primary outcome was within-subject coefficient of variation of K_i_ across two timepoints, expressed as a percentage. CV is the ratio of the within-subject standard deviation (SD_ws_) to the within-subject mean.

$$CV=SD_{ws}/Mean_{ws}\;\cdot 100$$ Eq. 1

However, to correctly calculate the mean CV for a group requires averaging the within-subject variances, rather than averaging CV estimated for each individual subject; this can be done by either a log-transformed or root mean square (RMS) approach, with similar results (M. Bland, 2006). In this dataset where negative values sometimes arise, a log-transformation would not be suitable and, therefore, we used the RMS method.

In many analyses, the within-subject standard deviation was positively correlated with the within-subject mean (tested with rank correlation coefficient), hence within-subject standard deviation could not be used as an index of measurement variability for method comparison. The repeatability coefficient (RC) was also reported (J. M. Bland & Altman, 1996) but not used as the primary outcome for the same reason.

$$RC=1.96\cdot \sqrt{SD_{ws}.}$$ Eq. 2

The intra-class correlation coefficient (ICC) was also reported using the two-way mixed effects, absolute agreement, and single rater model as recommended for a test–retest study (Koo & Li, 2016).

$$ICC\;=\;\frac{MS_{R}-MS_{E}}{MS_{R}+\left(k-1\right)MS_{E}}\;+\;\frac{k}{n}\left(MS_{C}-MS_{E}\right),$$ Eq. 3

where *k* = number of measurements, *n* = number of subjects, and *MS* = mean square for rows (*MS_R_*), columns (*MS_C_*), and error (*MS_E_*), from the analysis of variance (ANOVA) table. MATLAB code for ICC calculation used open-source tools developed by the University of Wisconsin-Madison (Molloy, 2016). Although ICC relates between-subject variance to total variance (and, therefore, within-subject variance) using the relationship shown in the equation, we are here interested in within-subject variation and not in between-subject variation. Moreover, whereas classically between-subject variance usually exclusively represents biological variation and within-subject variance usually exclusively represents analytical variation, in this study, biological variation is present both between and within subjects. For these reasons, CV and not ICC was used as the primary outcome.

Reference change values (RCV) were reported according to published methods to define the change in two within-subject serial measurements which can be considered significant (Fraser, 2011).

$$RCV=2^{1/2}\;\cdot \;\;Z\;\cdot \;\;{\left(CV^{2}\right)}^{1/2},$$ Eq. 4

where *Z* = the number of standard deviations for a desired probability (here set as 1.96 for 95%).

We could not strictly partition variability between biological and analytical sources but instead calculated the total variability and tested the effect of analysis factors. We did this in two ways: (1) *univariate*: modified a single analysis factor and tested the CV compared with the “default” analysis, by paired *t*-test, (2) *factorial*: generated results for all 256 possible combinations of analysis factors and ranked these according to CV. For both univariate and factorial tests, analysis factor combinations were only considered acceptable if meeting the biologically expected criteria defined above.

For the best performing analysis pipeline, further analyses examined the effect of age and inter-scan interval on variability, as well as tested for proportional bias using Bland–Altman plotting.

Analyses were performed in MATLAB and SPSS version 28 (IBM, Armonk, NY, United States).

## 3 Results

### 3.1 Participants

Thirty participants completed the study. All scans were reported by an experienced neuroradiologist, and none showed evidence of small vessel disease, neurodegenerative disease, or any other pathology (other than two small pineal cysts and one choroid fissure cyst).

Two subjects had intercurrent infection (one chest infection and one tonsillitis, both requiring antibiotics), and inter-scan intervals were extended per protocol to allow for recovery (28.3 and 30.2 weeks). However, on examination of individual CV values from the default method, both were clear outliers (Z-scores > 2). Both subjects were, therefore, excluded from further analysis on the suspicion of a biological effect on BBB permeability (Varatharaj & Galea, 2017). Therefore, 28 participants were included in the analysis (mean age 53.0 ± 5.8 years, age range 44–65, 82.1% female). Mean inter-scan interval was 9.9 ± 2.2 weeks (range 7.0–16.0 weeks). Mean (SD) time-of-day difference between paired scans was 101 (116) min.

Though the primary outcome of this study is the within-subject CV of K_i_, data on input functions and absolute K_i_ values are given below to aid interpretation.

### 3.2 Input functions

**Fig. 2.**
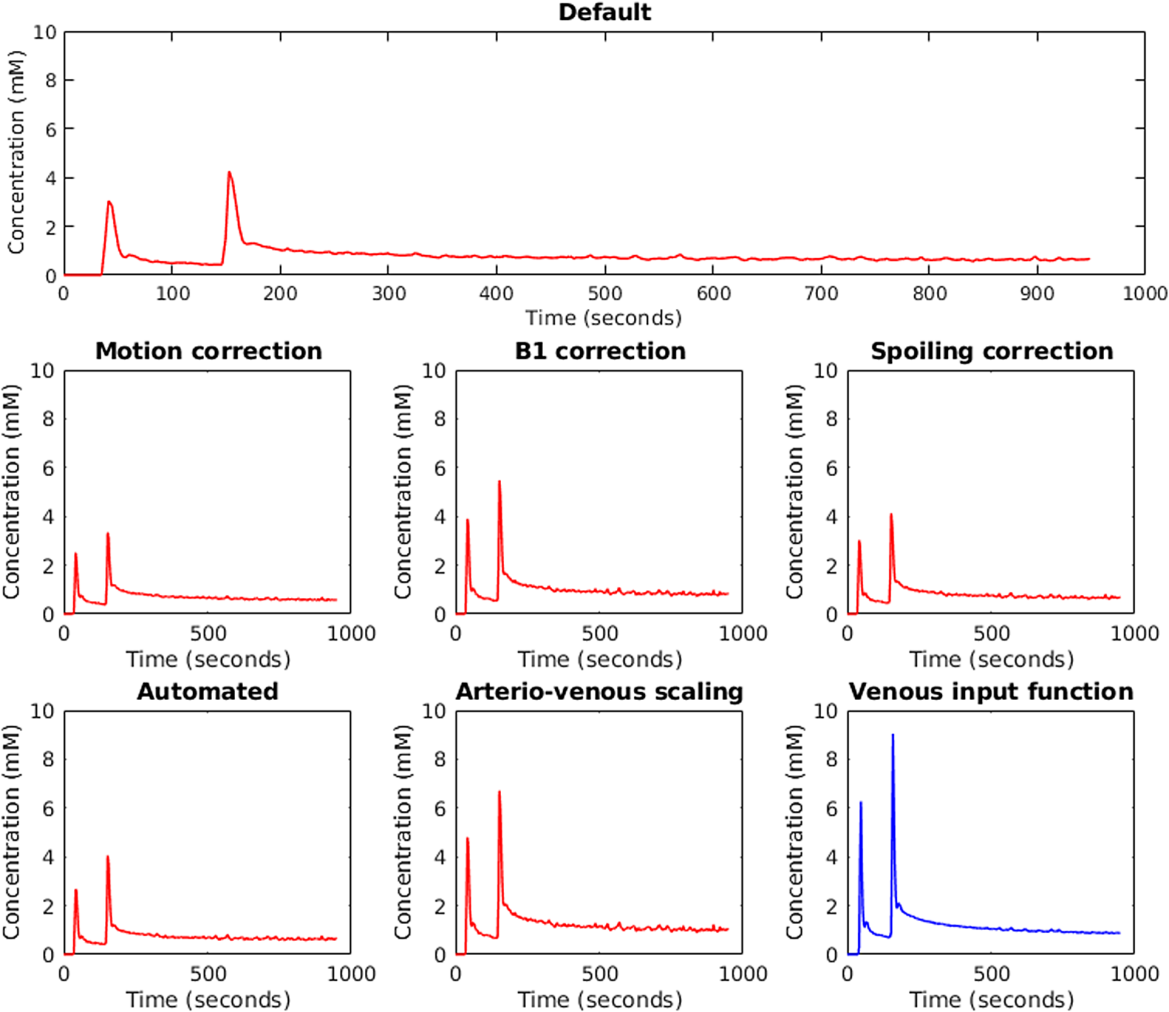
Input functions obtained from an example case using different analysis factors. Greater peak height when using the vein was significant in the whole group analysis.

Figure 2 shows input function examples obtained from a single participant when changing one analysis factor at a time. Across all scans for all participants, the manually delineated input function mask was significantly larger for the vein compared with the artery (21.45 ± 6.33 vs. 16.9 ± 5.6 voxels, p < 10^-4^, paired *t*-test for all comparisons in this section). Input function peak height was significantly higher for the vein (11.85 ± 5.15 vs. 8.46 ± 7.42 mmol/L, p = 0.007) but area under the curve (AUC) was not (1,146.13 ± 207.57 vs. 1,202.59 ± 672.33 s·mmol/L, p = 0.54). Within-subject CV for mask size was no different between artery and vein (25 vs. 27%, p = 0.80); however, within-subject CV for peak height and AUC was significantly lower for the vein compared with the artery (*peak height*: 32 vs. 59%, p = 0.005, *AUC*: 12 vs. 46%, p = 0.0002).

### 3.3 Absolute values, univariate testing

**Fig. 3.**
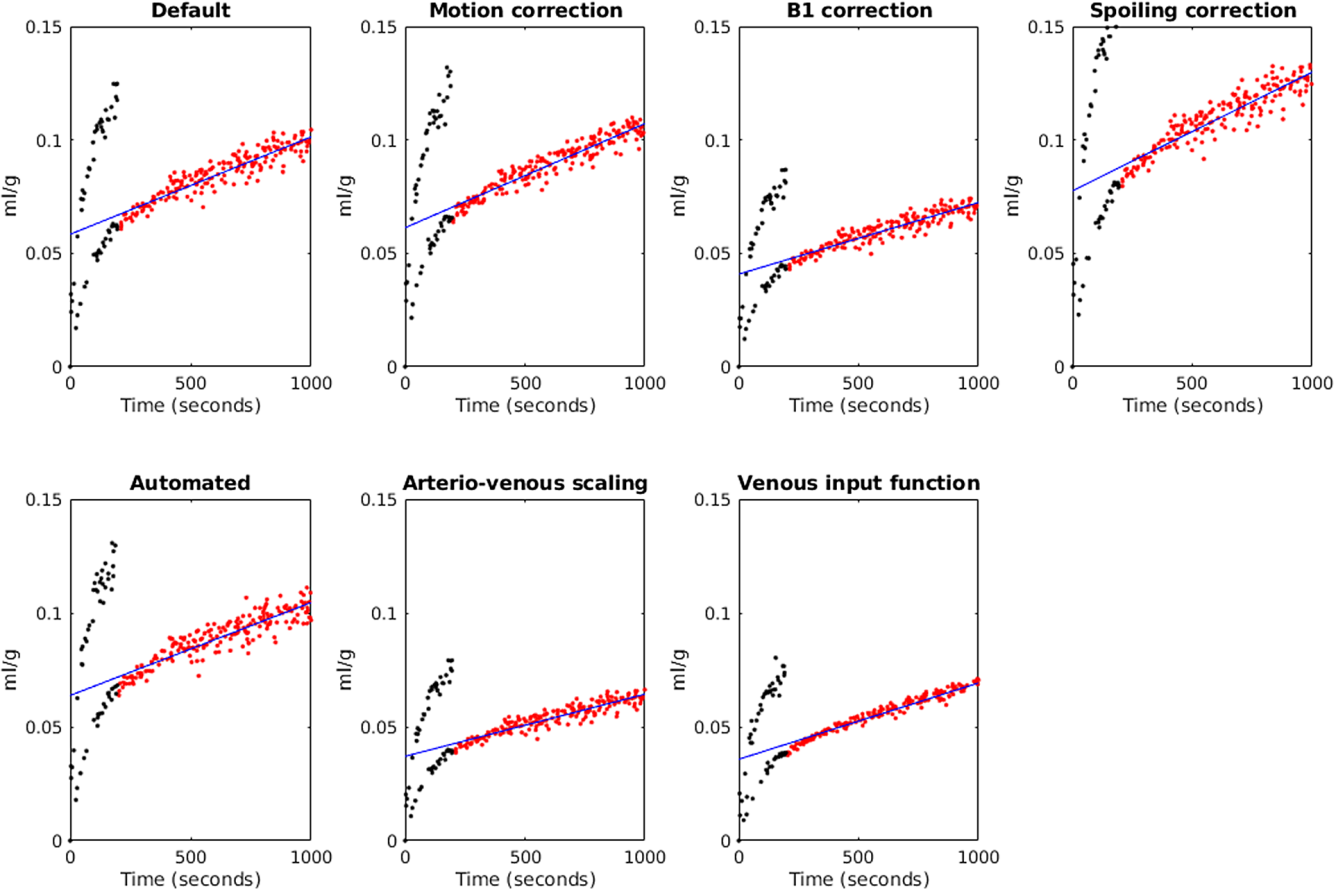
Patlak plots for white matter obtained from an example case (the same case as in Fig. 2) using different analysis factors. Red points are those included in the model, black points are excluded (rapid concentration changes during bolus injection), and the blue line is the fit.

Figure 3 shows Patlak plots for WM derived from an example case.

The effect of changing one analysis factor at a time on the absolute value of group mean K_i_ is reported in Table 1 (per-subject value is the mean of both visits). For WM, significant increases were seen with spoiling correction and the median, whilst a significant decrease was seen with B1 correction and input function scaling. Results for GM were similar except that the median gave a significant decrease rather than an increase.

**Table 1.** Effect of changing one analysis factor at a time on the absolute value of group mean K_i_.

	WM			GM		
Analysis	Group mean (SD) K_i_ (ml 100 g^-1^ min^-1^)	Mean (SD) difference in K_i_ compared with default (ml 100 g^-1^ min^-1^)	p-Value for comparison with default	Group mean (SD) K_i_ (ml 100 g^-1^ min^-1^)	Mean (SD) in K_i_ compared with default (ml 100 g^-1^ min^-1^)	p-Value for comparison with default
Default	0.055 (0.025)	n/a	n/a	0.094 (0.048)	n/a	n/a
Motion correction	0.136 (0.332)	0.081 (0.333)	0.22	0.110 (0.069)	0.012 (0.039)	0.12
B1 correction	0.046 (0.020)	-0.009 (0.008)	<0.001	0.085 (0.041)	-0.009 (0.011)	<0.001
Spoiling correction	0.057 (0.025)	0.002 (0.001)	<0.001	0.096 (0.048)	0.002 (0.001)	<0.001
Venous input function	0.051 (0.016)	-0.004 (0.021)	0.29	0.099 (0.037)	0.005 (0.045)	0.57
Automated input function	0.045 (0.029)	-0.010 (0.035)	0.14	0.084 (0.052)	-0.013 (0.069)	0.33
Input function scaling	0.043 (0.017)	-0.012 (0.023)	0.01	0.074 (0.036)	-0.020 (0.046)	0.03
Parameter-wise average	0.066 (0.039)	0.011 (0.028)	0.06	0.199 (0.290)	0.105 (0.266)	0.05
Median	0.058 (0.026)	0.003 (0.003)	<0.001	0.077 (0.037)	-0.017 (0.018)	<0.001

p-Values are by paired *t*-test. K_i_ results were compared with the pre-defined biologically expected criteria (Table 2). The criteria were met in all cases, except when using motion correction alone where the GM/WM K_i_ difference was lost (p = 0.42).

### 3.4 Variability in univariate testing

CV for the default analysis pipeline (as described in Methods) was 59% in WM and 53% in GM. The effect of changing one analysis factor at a time is reported in Table 3. The largest and most significant improvement was seen with the venous input function (*WM CV*: 30 vs. 59%, p < 0.001, *GM CV*: 21 vs. 53%, p < 0.001).

**Table 2.** Effect of changing one analysis factor at a time on the pre-defined acceptability criteria for K_i_, namely the ratio between white matter (WM) and grey matter (GM), and the relationship with cerebral blood volume (CBV) and flow (CBF).

Analysis	Within-subject GM/WM K_i_ ratio		β (p-value) for CBV to predict K_i_
	Mean (SD)	p-Value	
Default	1.82 (0.64)	<0.001	0.73 (<0.001)
Motion correction	1.37 (0.93)	0.42	0.42 (0.03)
B1 correction	1.97 (0.71)	<0.001	0.75 (<0.001)
Spoiling correction	1.79 (0.64)	<0.001	0.71 (<0.001)
Venous input function	2.10 (0.67)	<0.001	0.79 (<0.001)
Automated input function	1.77 (0.63)	<0.001	0.78 (<0.001)
Input function scaling	1.83 (0.64)	<0.001	0.70 (<0.001)
Parameter-wise average	2.80 (2.15)	0.01	0.74 (<0.001)
Median	1.42 (0.45)	<0.001	1.05 (<0.001)

In the step-wise regressions, CBF did not improve the variance explained and so was not included in any model. The p-value is for a paired *t*-test comparing within-subject GM and WM K_i_ values.

**Table 3.** Variability results obtained in univariate testing (changing one analysis factor at a time).

	WM			GM		
Analysis	CV (%)	p-Value for comparison with default	ICC	CV (%)	p-Value for comparison with default	ICC
Default	59	n/a	0.01	53	n/a	0.06
Motion correction	61	0.74	0.03	53	0.57	0.23
B1 correction	58	0.36	0.06	52	0.14	0.08
Spoiling correction	57	0.03	0.01	53	0.01	0.07
Venous input function	30	<0.001	0.61	21	<0.001	0.80
Automated input function	52	0.30	0.55	40	0.04	0.49
Input function scaling	34	0.001	0.66	30	0.002	0.68
Parameter-wise average	60	0.37	0.08	58	0.16	0.08
Median	58	0.53	0.03	51	0.27	0.05

p-Values are by paired *t*-test. ICC = intra-class correlation coefficient (two-way mixed effects, absolute agreement, single rater model).

### 3.5 Variability in factorial testing

**Fig. 4.**
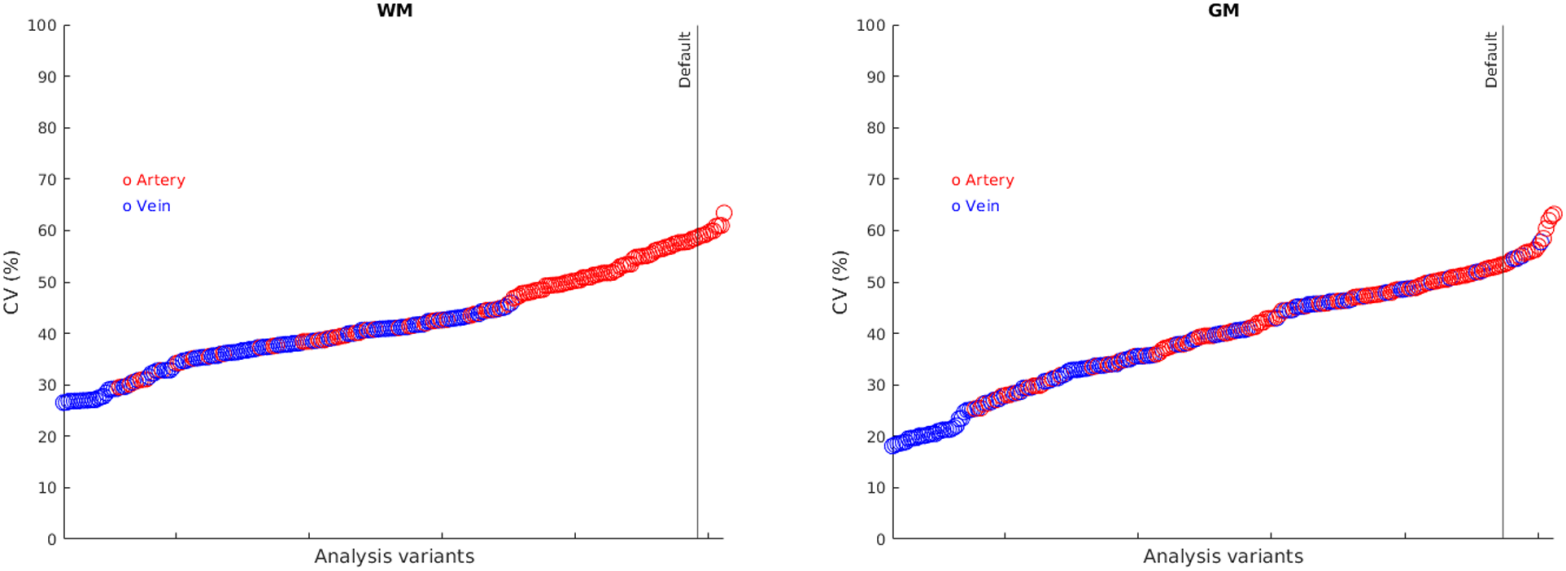
Ordering of analysis factor combinations according to coefficient of variation (CV). Those using the artery are shaded red, those using the vein are shaded blue. The vertical line indicates the “default” method. The methods with lowest CV are generally those using the vein.

Analysis factor combinations were ordered according to CV, as shown in Figure 4. Use of the vein clearly dominated improvements in CV.

The combination of analysis factors with optimal CV for both WM and GM is shown in Table 4, giving CV of 27% and 17% for WM and GM, respectively. The pre-defined biological criteria were met: WM/GM ratio 1.49 ± 0.46 (mean ± SD), p < 0.001 (one-sample *t*-test comparing ratio to 0), β for CBV for predict K_i_ = 0.63, p < 0.001, CBF not included in model). For WM, the “best” method did not affect absolute K_i_ (0.057 vs. 0.055, p = 0.74) and improvement in CV compared with default (27% vs. 59%, p < 0.001). For GM, the “best” method gave a trend to decrease absolute K_i_ (0.078 vs. 0.094, p = 0.06) and an improvement in CV (17% vs. 53%, p < 0.001).

**Table 4.** Details of analysis pipeline with lowest coefficient of variation (CV), obtained from factorial testing of all combinations of analysis factors.

Factor	Default		Best	
Motion correction	Off		**On**	
B1 correction	Off		Off	
Spoiling correction	Off		Off	
Input function location	Artery		**Vein**	
Input function method	Manual		Manual	
Input function scaling	Off		**On**	
Averaging level	Signal-wise		Signal-wise	

Averaging method	Mean		**Median**	
	*WM*	*GM*	*WM*	*GM*
Mean (SD) K_i_ (ml/100 g/min)	0.055 (0.025)	0.094 (0.048)	0.057 (0.016)	0.078 (0.025)
CV (%)	59	53	27	17
Reproducibility coefficient (ml/100 g/min)	0.096	0.175	0.036	0.039
Reference change value (%)	164	147	75	47
ICC	0.01	0.06	0.50	0.73

Changes from the default method are shown in bold. ICC = intra-class correlation coefficient (two-way mixed effects, absolute agreement, single rater model).

There was little difference in CV between the highest ranking analysis factor combinations. A small and non-significant further improvement in CV could be obtained for WM by using B1 correction (26 vs. 27%, p = 0.47), and for GM by using spoiling correction (15 vs. 17%, p = 0.74).

**Fig. 5.**
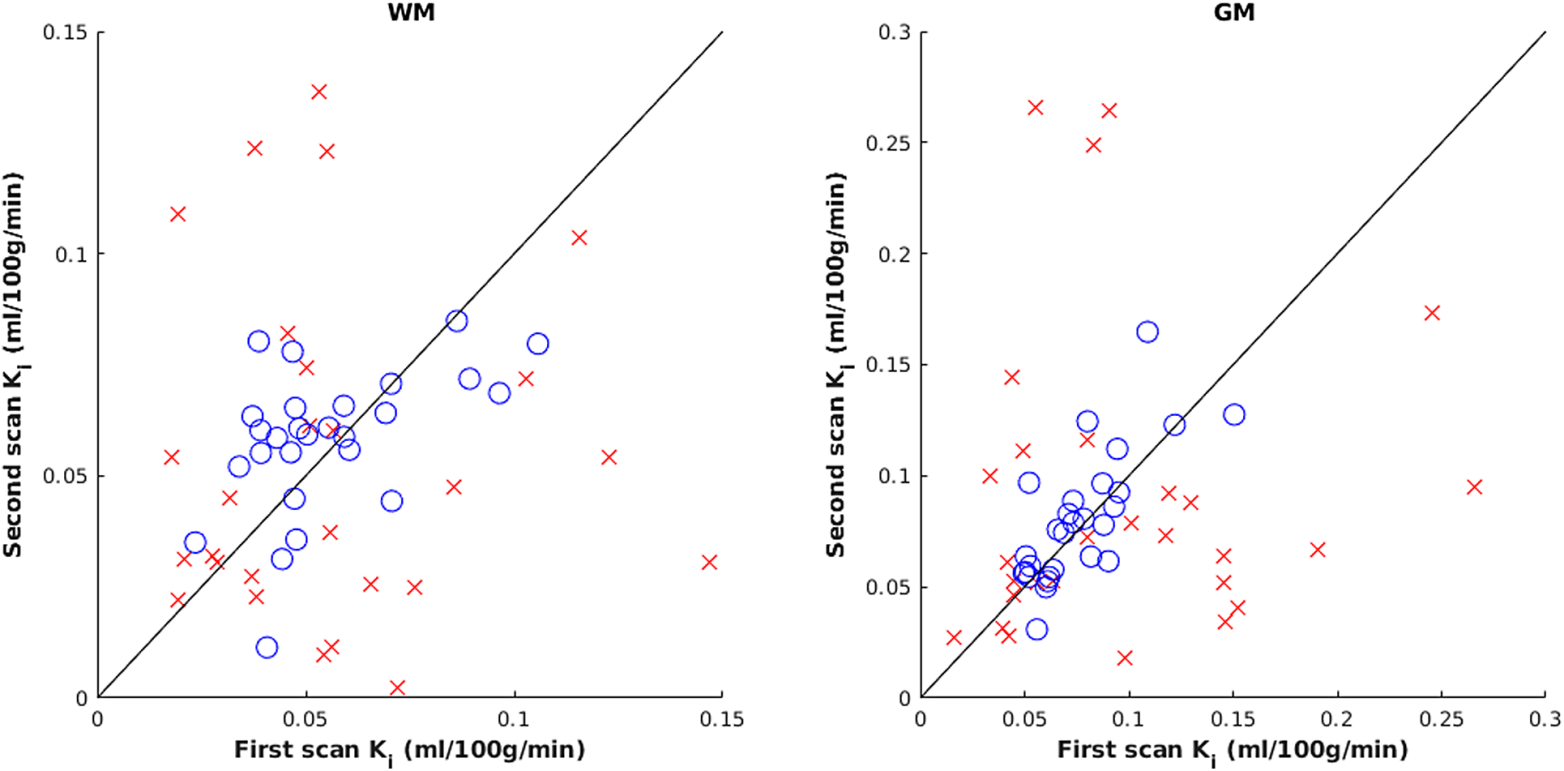
Scatter plot showing K_i_ values for each subject obtained at first and second timepoints, with line of equality, for the default (red crosses) and “best” methods (blue circles).

Plotting of per-subject K_i_ values for first and second scans was used to visualise the improved absolute agreement of within-subject values (see Fig. 5). With the default method, there was no correlation between paired within-subject values (WM: p = 0.87, Kendall’s τ = -0.03, GM: p = 0.38, τ = 0.12); however, using the “best” method a significant positive correlation emerged (WM: p = 0.01, τ = 0.34, GM: p < 0.001, τ = 0.55). In a one-tailed William’s test, p-values for the improvement in correlation were 0.09 and 0.04 for WM and GM, respectively.

### 3.6 Differences in variability between subjects

The analysis method ranked best for CV was taken forward to test for differences in variability between subjects. There was no correlation between CV and age (*WM*: p = 0.82, τ = 0.03, *GM*: p = 0.48, τ = -0.01). There was no correlation with inter-scan interval in WM (p = 0.49, τ = -0.10), though there was a trend in GM (p = 0.07, τ = -0.25). There was no correlation between time-of-day difference and CV (WM: p = 0.71, τ = 0.08, GM: p = 0.81, τ = -0.05). There was no effect of gender (WM: p = 0.77, GM: p = 0.83).

**Fig. 6.**
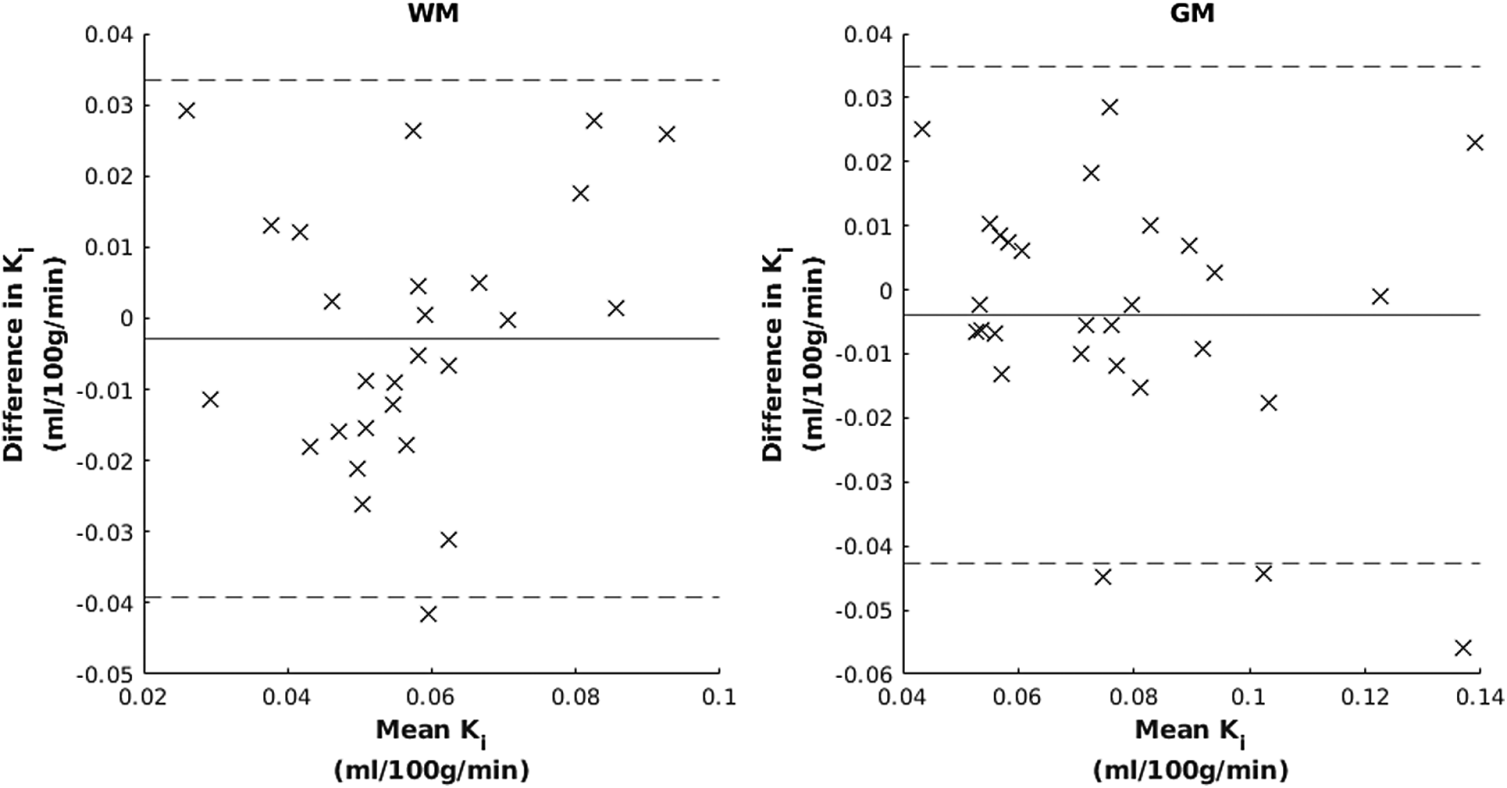
Bland–Altman plots for white matter (WM) and grey matter (GM). The horizontal lines show mean difference and 95% limits.

Bland–Altman plots showed no evidence of proportional bias (Fig. 6), and correspondingly there was no correlation between means and differences of K_i_ over scan sessions (WM: p = 0.28, τ = 0.15, GM: p = 0.23, τ = -0.16). The 95% limits of agreement were 0.073 and 0.078 ml/100 g/min for WM and GM, respectively.

### 3.7 Effect of removing input function variability

Repeating the highest ranked method but replacing the subject-derived input function with one standard input function derived from an external population did not significantly affect CV (*WM*: 26.8 vs. 26.6%, p = 0.08, *GM*: 14 vs. 17%, p = 0.16).

### 3.8 Effect of dynamic sequence duration

**Fig. 7.**
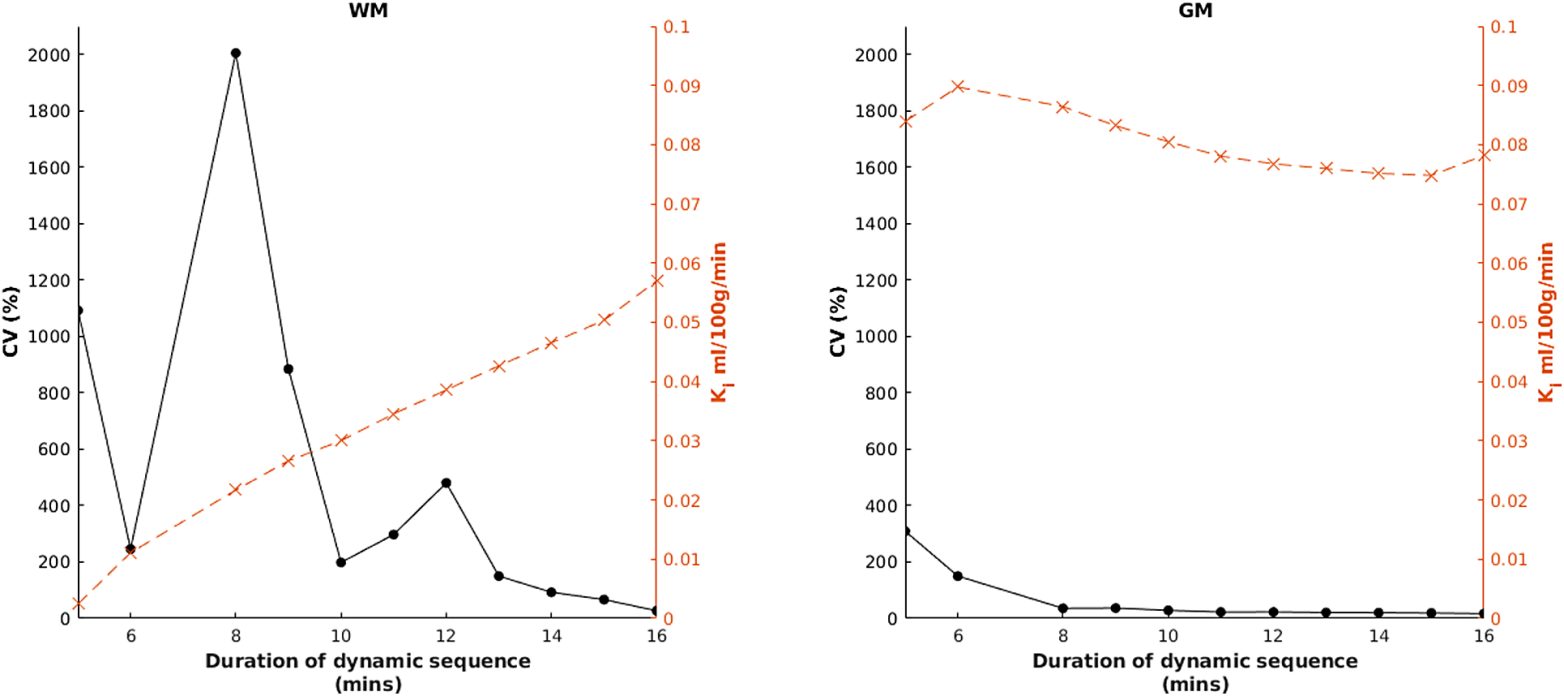
Effect of dynamic sequence duration on the coefficient of variation (CV, black circles, solid line) and K_i_ (orange crosses, dashed line) for both white matter (WM) and grey matter (GM). Data for the 7-min timepoint are not shown, as due to a high proportion of negative K_i_ estimates, summary statistics become highly unstable.

Figure 7 shows results obtained by repeating the highest ranked method but with the dynamic sequence truncated to 1-min increments between 5 and 16 min (i.e., restricting the amount of post-contrast data available). The deterioration in CV was most marked in WM.

## 4 Discussion

### 4.1 Summary

In this study, we performed scan–rescan measurements using brain DCE-MRI in 30 adults with an interval of approximately 10 weeks. We found agreement with biological expectations of K_i_ as a compound marker of permeability and surface area, with higher values in GM compared with WM.

Relatively small changes in absolute values occurred with analysis factors such as B1 and spoiling correction and using the median instead of the mean. In contrast, measurement variability was highly dependent on analytical factors, predominantly related to input function selection. Using the vein as input function was the single most effective parameter in terms of improving CV. Through factorial testing, we identified the optimal combination of factors, giving a CV of 27 and 17% in WM and GM, respectively. There was no evidence of proportional bias, and we did not identify any subject factors associated with variability. Finally, we showed a significant effect on dynamic sequence duration, both on variability and absolute K_i_ values. The results from this study will inform analysis methods and sample size calculation for future studies using DCE-MRI.

### 4.2 Interpretation

Our finding of a precision benefit with using the vein supports published guidance (Thrippleton et al., 2019) and recent experimental data (Lewis et al., 2022). We found that the artery had lower peak height, suggesting contamination by inflow artefact. Arterial regions-of-interest were also smaller, giving greater susceptibility to partial volume artefact. Both reasons likely contributed to the finding of significantly greater inter-scan variation (in AUC) of input functions derived from the artery compared with those from the vein. Since the subject-derived input function is a major source of uncertainty (Garpebring et al., 2013), it is reasonable to consider replacing individual input function measurement with a population standard; however, in this study we did not find this to give any significant precision improvement over that of using the vein. Also, another recent study showed no significant impact of input function selection on variability (Cramer et al., 2023). The findings of our study suggest that the precision benefit of using the vein is strongly technique dependent; in this case likely reflecting the differences between our 3D acquisition and the reported 2D protocol, where slice placement for the input function is carefully optimised for the flow direction of the ICA (Cramer et al., 2023). However, extending these findings from a relatively homogeneous healthy control group to subjects with pathology requires caution, as there is likely to be greater inter-individual variation in the true input function, which if not included in the model fit may obscure important differences (Keil et al., 2017). Novel approaches have been suggested for compromise, such as normalising each individual input function to match the area under the curve of a population standard (Kleppesto et al., 2022).

We did not find a precision improvement with B1 mapping despite this often being mentioned as an important component of T1 mapping by the VFA method (Bane et al., 2018). This could be an issue with our choice of B1 mapping technique, though we chose a validated method (Chung et al., 2010) which we also tested with a phantom on our scanner (data not shown). It is important to consider that whilst B1 mapping might improve the *accuracy* of parameter estimation (Manning et al., 2021), the additional noise introduced by B1 mapping could actually reduce overall precision (Lee et al., 2017). Indeed, this is true for many of the factors, as this study examined only precision not accuracy.

We also showed a marked effect of dynamic sequence duration on variability, as has been previously reported (Cramer & Larsson, 2014; Wong et al., 2017). This is likely due to an increased signal-to-noise ratio (SNR) obtained with greater tissue concentrations at longer durations, and hence is most prominent in WM.

Though we could not partition variability between biological and analytical sources, we can say that analytical imprecision must be less than the total variability of 17–27%. To what degree biological variation is relevant remains unknown, but inferences can be drawn from the literature. Firstly, since K_i_ is also determined by the vascular surface area (which is indirectly reflected by CBV), biological variation in CBV will also contribute to variation in K_i_ measurements. Changes in CBV may occur in the context of functional activation (Krieger et al., 2012), which could easily vary between scanning sessions. Using measured data and the regression model, a 1 ml/100 g increment in microvascular volume such as might occur in the occipital cortex during visual stimulation (Krieger et al., 2012) would give a CV of 14% for GM K_i_ between two sessions, that is, comparable with the observed CVs. Secondly, although the study protocol sought to avoid the effect of intercurrent infections, asymptomatic fluctuations in inflammatory state are not uncommon (Stuart et al., 2020) and could contribute to variability in BBB permeability (Varatharaj & Galea, 2017).

Another study of variability in brain DCE-MRI reported lower CV (12 and 14% in WM and GM), though the inter-scan interval was much shorter (typically 1 day) and negative K_i_ values were removed (Wong et al., 2017). It also appears that the CV in this study was calculated using the group mean, which may give an underestimate (M. Bland, 2006). A comparable study reported a better ICC for WM (0.79) and similar for GM (0.71), though again the interval was much shorter than here (1 week) and the population significantly younger (Cramer et al., 2023). Although we did not see overt changes of cerebrovascular disease in any of our subjects on conventional imaging, age-related BBB disruption (Montagne et al., 2015) would be expected to increase biological variability. However, we did not find an effect of age on variability within our cohort.

Other studies have reported higher variability in GM compared with WM (Cramer et al., 2023; Wong et al., 2017), hypothesised due to a greater susceptibility to partial volume artefact from CSF and vessels, especially in cortical GM. However, we found the opposite, a lower CV in GM. Since signal changes were summarised by the median within a strict tissue mask, our method is likely to have been robust to outliers. However, this does not explain why those same factors failed to further improve CV in WM. Since the absolute value of K_i_ is lower in WM, one would expect a lower tissue concentration and, therefore, a lower SNR than in GM. The greater noise contribution would contribute to greater analytical imprecision in WM compared with GM. Also, radiofrequency penetration effects are likely to give greater B1 inhomogeneity in WM than cortical GM, which (assuming imperfect correction) would contribute to imprecision. There is also the possibility of tissue differences in biological variability, though this study cannot comment on these.

We have avoided specifying a cutoff value for measurement variability since this is dependent on the use case; instead, we present our findings to be appraised by users of DCE-MRI. However, it is relevant to mention the measurement variability of some other physiological parameters, to place these values in context. Considering another technique assessing the cerebral vasculature, CBF measured by phase contrast MRI has a CV of 4–15% (in younger and older subjects, respectively) (Ismaili et al., 2018). Thinking of a much simpler and more established method, the CV of blood pressure is 10–17% (Marshall, 2008). Of course, each of these studies has differences in patient population, measurement interval, and biological variability of the measurand, but the values give an idea of what is considered an “acceptable” measurement variability for other widely used techniques. For researchers, the adequacy of the CV is relative to the sample size and expected effect size in a given study, and users can and employ our results for this purpose.

We quote the RCV since this is widely used for reporting measurement variability of laboratory analytes, and, therefore, places our data in a wider context. For example, C-reactive protein (CRP) measurement is used ubiquitously in clinical practice and analytical imprecision for a widely used automated system is 1–5% (Zimmerman et al., 2015). However, biological variation in CRP is 34% (European Federation of Clinical Chemistry and Laboratory Medicine, n.d), giving an RCV of 94–95%. This compares favourably with the RCVs for the optimised method reported here.

### 4.3 Strengths

This study had a good sample size in comparison with other scan–rescan studies in the field, and adherence to the pre-defined protocol was robust. There was no missing data, and except for two outliers with a plausible biological explanation, all data were included in the final analysis.

In terms of absolute values, K_i_ reported here is similar to healthy control values measured by PET (Iannotti et al., 1987) using 68-Ga-DTPA, a hydrophilic compound with comparable molecular weight to Gadovist (440 vs. 604 Daltons). A study using a comparable protocol at a different site reported similar values for segmented GM though lower values for WM (Cramer et al., 2023). However, other studies have reported values an order of magnitude lower (Montagne et al., 2015), and some of the factors covered in this study may explain this, if they differed between centres. Inter-centre differences and possible explanations have been reviewed in detail elsewhere (Raja et al., 2018) and there are ongoing efforts to standardise acquisition and analysis methods (Thrippleton et al., 2019). In particular, signal drift may significantly affect results (Heye et al., 2016), and better methods for detection and correction are needed.

Underestimation of the arterial signal due to inflow artefact would be expected to overestimate parameter values (Lavini, 2015), and we did find lower absolute K_i_ values when using the vein (though not a significant difference).

### 4.4 Weaknesses

The study design was pragmatic; however, the addition of further timepoints (including one with a much shorter interval) would have helped to pick apart biological variation from analytic imprecision. However, this was weighed against burden and acceptability to participants.

Our acceptability criteria for K_i_ values were determined by biological expectations. However, our assumption that higher vascular density in GM (Lierse & Horstmann, 1965) should lead to higher surface area and, therefore, higher K_i_ may be overly simplistic. Recent evidence suggests that WM vessels, though on average less dense, tend to be wider (Hase et al., 2019); this would tend to cancel out the effect of density on surface area. There is also considerable regional heterogeneity in vascular architecture (Lierse & Horstmann, 1965) and transcriptome (Bryant et al., 2023), raising the possibility that regional differences in both permeability and surface area may be too granular to reasonably use a large tissue mask to characterise an entire tissue. Also, one needs to consider the reduction in vascular density that occurs with ageing (mean age of study population here was 53 years), and whether this could skew the WM/GM ratio; there is evidence to suggest that the rate of loss in healthy individuals (without vascular dementia) is greater in the cortex than in the white matter (Brown et al., 2007; Leenders et al., 1990).

The study population was 82% female. There is evidence that sex hormone levels influence BBB permeability (Wilson et al., 2008), and we did not account for either menstruation or menopause in our study, though there was no effect of gender on variability.

There are many other physiological factors which could potentially influence BBB permeability, which we did not control for in this study. For example, subclinical systemic inflammation (Varatharaj & Galea, 2017), caffeine intake (Chen et al., 2010), and physical exercise (Malkiewicz et al., 2019).

Others have examined the causes of systematic errors in DCE-MRI (Manning et al., 2021), and our protocol did exclude early post-injection data as suggested. However, we used a bolus instead of a slow injection, as we wished to also measure CBF in the same session. Our slice thickness (5 mm) was relatively large, reflecting a compromise between the desired spatial and temporal resolutions, coverage, and SNR.

## 5 Conclusion

In conclusion, this study provides data on measurement variability of brain DCE-MRI over a time frame applicable to clinical and research use. The metrics reported here can be used for sample size calculation and to guide judgement of a significant effect in longitudinal assessments.

## Data and Code Availability

Data and code are subject to ongoing analyses and available upon reasonable request from a qualified investigator.

## Author Contributions

A.V.—conceptualisation, data curation, analysis, investigation, software, writing (draft and review). C.J.—investigation, writing (review). A.D.—supervision, resources, writing (review). B.Y.—analysis, writing (review). S.C.—software, writing (review). H.L.—software, supervision, writing (review). I.G.—conceptualisation, supervision, writing (review).

## Funding

This study was funded by the Medical Research Council (MRC: MR/R017352/1) and National Institute for Health and Care Research.

## Declaration of Competing Interest

The authors declare no potential conflicts of interest relevant to this work.

## Supplementary Materials

Supplementary material for this article is available with the online version here: https://doi.org/10.1162/imag_a_00324

## Supplementary Material

Supplementary Material
