# Supplementary material for: Measurement variability of blood–brain barrier permeability using dynamic contrast-enhanced magnetic resonance imaging
Source: Imaging Neurosci (Camb). 2024 Oct 22;2:imag-2-00324. doi: 10.1162/imag_a_00324 (PMC11497077; doi:10.1162/imag_a_00324)
Supplement: Supplementary Material [file imag_a_00324-supp.pdf]

**Voxels with negative Ki values**

This is only applicable to the parameter-wise method, where $K_i$ was calculated for every voxel individually. In this context, there was a small but significant reduction in the percentage of negative values when using the vein instead of the artery (12.9% vs 14.9%, $p < 0.001$). The factor which gave a marked reduction in negative values was motion correction (8.2%, $p < 0.001$ compared to default). With the 'best' method from the main paper, there was further reduction to 5.7% ($p < 0.001$ compared to default).

| **Analysis (all using parameter-wise averaging)** | **Proportion of negative values (mean % and SD)** | **P-value for comparison to default** |
|---|---|---|
| Default | 14.9 (8.0) | n/a |
| Motion correction | 8.2 (8.0) | <0.001 |
| B1 correction | 15.9 (8.0) | 0.33 |
| Spoiling correction | 15.1 (8.0) | <0.001 |
| Venous input function | 12.9 (8.0) | <0.001 |
| Automated input function | 15.4 (10.1) | 0.56 |
| Input function scaling | 14.9 (8.0) | 0.99 |
| 'Best' method | 5.7 (6.1) | <0.001 |

Supplementary Table 1: Proportion of negative values, mean for all subjects per analysis method. All methods in this Table use parameter-wise averaging of $K_i$ values calculated voxelwise. Hence the 'Default' and 'Best' methods presented here are not the same as those presented in the main paper, which used signal-wise averaging (however, all the other factors are the same).

**Example vascular regions of interest**

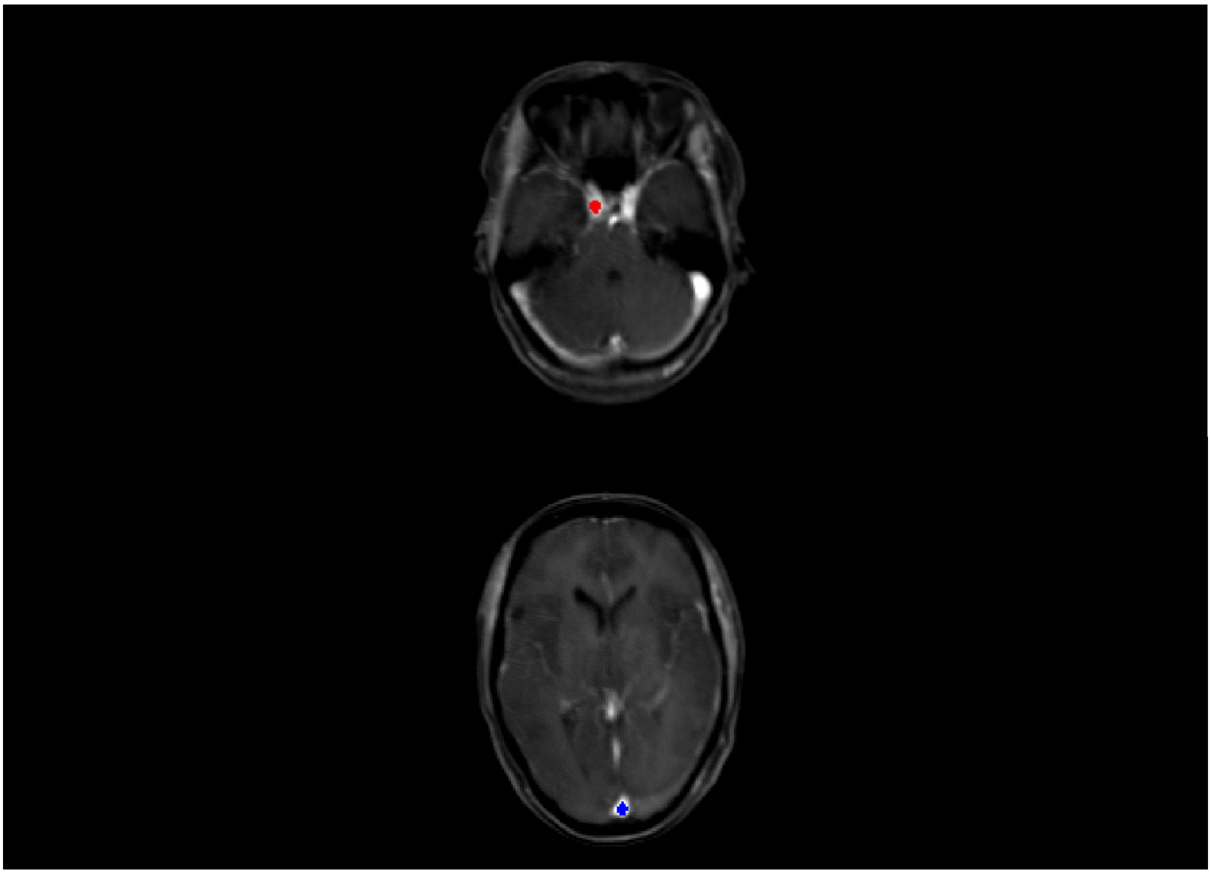

Supplementary Figure 1: Vascular regions of interest created manually for input function extraction from a single example case (the same case as in Figure 2 and 3 in the main paper). The top image shows the artery (red), the bottom the vein (blue).

**Example voxelwise $K_i$ maps**

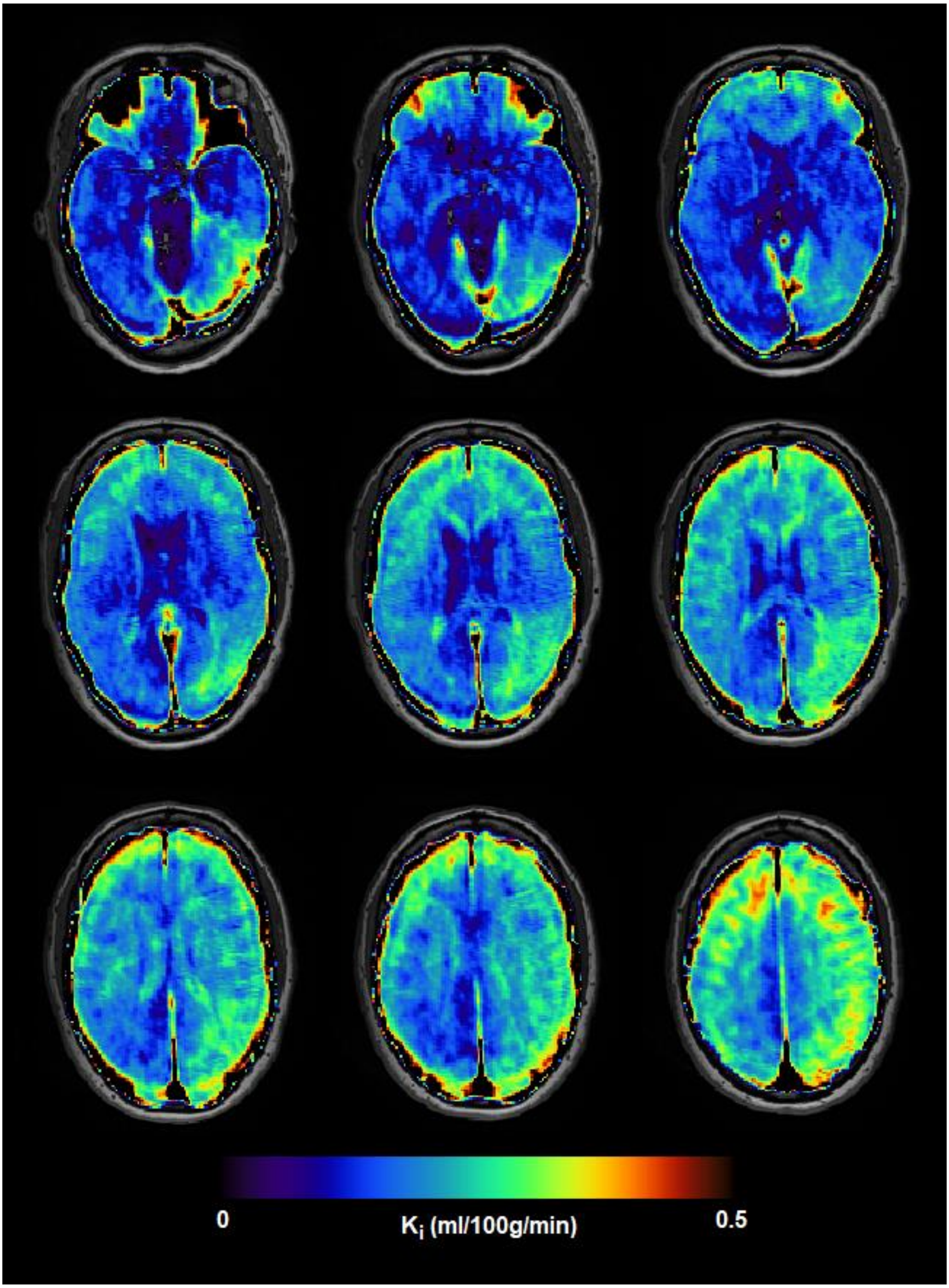


Supplementary Figure 2: Voxelwise $K_i$ map, created using the 'best' method with parameter-wise calculation, and overlaid on the T1 image. This is the same case which has been used an example throughout.
